# Multidrug use positively correlates with high-risk prescriptions in the Japanese elderly: a longitudinal study

**DOI:** 10.1186/s40780-019-0150-6

**Published:** 2019-09-02

**Authors:** Sayaka Arai, Takahiro Ishikawa, Hisaya Kato, Masaya Koshizaka, Yoshio Maezawa, Takako Nakamura, Takaaki Suzuki, Koutaro Yokote, Itsuko Ishii

**Affiliations:** 1 Pharmacy of Chiba University Hospital, Chiba, Japan; 2 Geriatric Medical Center, Chiba University Hospital, 1-8-1 Inohana, Chuo-ku, Chiba City, Chiba, 260-8677 Japan; 3 Endocrinology, Hematology and Gerontology, Chiba University Graduate School of Medicine, Chiba, Japan; 4 Department of Diabetes, Metabolism and Endocrinology, Chiba University Hospital, Chiba, Japan

**Keywords:** Elderly, Hospitalization, Polypharmacy, Potentially inappropriate medications

## Abstract

**Background:**

There is a lack of evidence that multidrug use triggers adverse events. Therefore, the main purpose of this study was to clarify the relationship between the total number of drugs and number of high-risk prescriptions administered to Japanese elderly patients.

**Methods:**

Using hospital electronic medical records (EMR), we evaluated the prescriptions of outpatients aged 65 years or older. We defined prescriptions of potentially inappropriate medications (PIMs) and overlapping prescription of drugs with the same mechanism of action (DSAs) as high-risk prescriptions. We analyzed the relationship among total number of drugs and high-risk prescriptions. In addition, we performed a secondary research to determine whether the hospitalization rate and concomitant medication contents differ depending on the high-risk prescriptions.

**Results:**

Data for 13,630 outpatients were analyzed. A significant positive correlation between the numbers of total drugs and PIMs was found. The prescription frequency of individual PIMs rose as the total number of prescription drugs increased. The odds ratio (OR) of overlapping DSAs was significantly higher in patients using 5 or more drugs. In addition, there were significantly more prescriptions of laxatives among patients with overlapping prescriptions of anticholinergic drugs. The use of almost all PIMs was not an independent risk factor for hospitalization; instead, the number of PIMs was an independent risk factor for hospitalization [OR 1.18 (95% CI, 1.12–1.26)].

**Conclusions:**

The number of PIMs and overlapping DSAs were high in patients receiving multidrug treatment. To avoid adverse events and hospitalization, it might be useful to review prescriptions and consider the number of PIMs and overlapping DSAs.

## Background

Multidrug use, often termed “polypharmacy,” has negative consequences [1, 2]. However, there is a lack of evidence that multidrug use triggers adverse events. Some reports suggested a relationship between the number of drugs and lower rate of adherence [3], risk of potential drug-drug interactions [4], and inappropriate prescriptions, such as potentially inappropriate medications (PIMs).

PIMs pose a high risk of adverse events in the elderly and should be avoided. Two sets of criteria for PIMs, the Beers criteria [5] and the Screening Tool of Older Persons’ Prescriptions (STOPP) [6, 7], are used globally. In Japan, the “Screening Tool for Older Persons’ Appropriate Prescriptions for Japanese (STOPP-J) [8]” is used. The use of drugs related to the Beers or STOPP criteria and the consequent under-prescribing of medications were reported to be frequent in patients receiving multidrug treatment [9–11]; similar reports were also published in Japan [12, 13]. We hypothesize that adverse events tend to occur in patients receiving multidrug treatment because of high-risk prescriptions. However, to date, a detailed analysis from this perspective has not been performed.

This study aimed to clarify the relationship between total number of drugs and high-risk prescriptions administered to Japanese elderly patients, and to clarify whether hospitalization rate and concomitant medication differ based on high-risk prescriptions used by these patients.

## Methods

### Survey method and subjects

This survey was conducted at Chiba University Hospital, which has 37 clinical departments conducting consultations for an estimated 2500 outpatients/day. Using the electronic medical records (EMR) system at this hospital, we surveyed prescriptions for outpatients aged ≥65 years who were presented at this hospital between October–December 2016 and prescribed at least one regular medication. All regular medications except for as-needed medications, injections, and topical drugs were analyzed.

### Examination of prescriptions and hospitalization

We extracted 21 categories from the list of drugs that should be prescribed with special caution in the STOPP-J; these were defined as PIMs. In the STOPP-J, eight categories had limited disease and patient background, which were excluded from PIMs because it was impossible to extract their data from the EMR using our method.

To find overlapping of drugs with the same mechanism of action (DSAs), we surveyed drugs with anticholinergic effects (39 drugs) and benzodiazepine receptor agonists (22 drugs). DSAs include drugs with different medical effects. Because constipation is a typical adverse event of anticholinergic action, we considered that laxative prescription may be an indicator for the occurrence of adverse events.

We defined prescriptions of PIMs and overlapping prescription of DSAs as high-risk prescriptions. We examined the relationship between the number of drugs and high-risk prescriptions. We also investigated the number of hospitalizations that occurred for one year following the prescription survey (January to December 2017).

### Statistical analysis

To compare the median values of PIMs, the Mann-Whitney U test was performed following the Kruskal-Wallis test. Correlation between the total number of prescribed drugs and number of PIMs was obtained using the Spearman’s rank correlation coefficient. For the prescription status of PIMs and overlapping DSAs, we performed a χ^2^ test or Fisher’s exact test, as appropriate. Logistic regression analysis was performed on the prescription of PIMs and the effect of the number of PIMs on hospital admission. All analyses were carried out using IBM SPSS Statics ver. 23 (IBM Corp., Armonk, NY). Significance was set to 5%, and correction for multiple comparisons was conducted using the Bonferroni method.

## Results

**Fig. 1:**
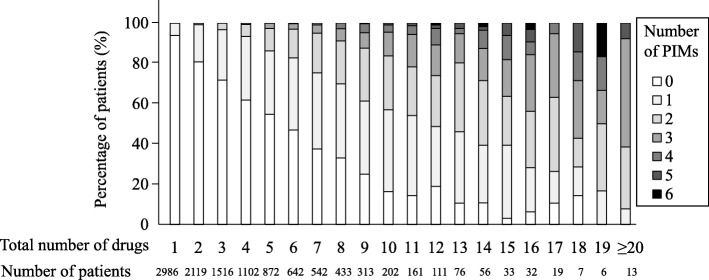
Relationship between the total number of drugs and the number of PIMs. Prescription ratio of PIMs by total number of drugs

Patient characteristics are shown in Table 1. A total of 11,241 elderly outpatients received at least one regular medication. As the total number of drugs increased, the prescription frequency of PIMs also increased (Table 2). For 17 of the 21 PIM categories, prescription frequency was significantly higher in the 5–9-drugs group than in the 1–4-drugs group. It was similar in the 10 or more-drugs group, compared to the 1–4-drugs group. A significant correlation was found between the number of PIMs and total number of prescribed drugs ((Fig. 1, Spearman’s *r* = 0.529, *p* < .001). The median number of PIMs was 0, 1, and 2 in the 1–4-, 5–9-, and 10 or more-drugs groups (*p* < .001 in each case), respectively.

**Table 1:** Patient Characteristics

Characteristics	All categories	1–4 drugs	5–9 drugs	≥10 drugs
	*n* = 11,241	*n* = 7723	*n* = 2802	*n* = 716
	(% or range)	(% or range)	(% or range)	(% or range)
Sex				
Male	5653 (50.3)	3765 (48.8)	1430 (51.0)	393 (54.9)
Female	5588 (49.7)	3958 (51.2)	1372 (49.0)	323 (45.1)
Median age (years)	73 (65–100)	73 (65–98)	73 (65–95)	73 (65–100)
Age group				
65–74 years	6444 (57.3)	4384 (56.8)	1646 (58.7)	414 (57.8)
≥ 75 years	4797 (42.7)	3339 (43.2)	1156 (41.3)	302 (42.2)
Median number of prescribed drugs	3 (1–25)	2 (1–4)	6 (5–9)	11 (10–25)
Median number of consultation departments^†^	1 (1–6)	1 (1–3)	1 (1–5)	2 (1–6)

†The Mann-Whitney U test was used after the Kruskal-Wallis test to compare the differences among groups. Correction by the Bonferroni method was performed, and *p* < .017 was considered statistically significant. In the comparison of medians between the 1–4-drugs and 5–9-drugs groups, the 1–4-drugs and 10 or more-drugs groups, and the 5–9-drugs and 10 or more-drugs groups, each *p* value was < .001

**Table 2:** Prevalence of drugs that should be prescribed with special caution

PIMs (Drug class or generic names)	All categories *n* = 11,241	1–4 drugs *n* = 7723 (%)	5–9 drugs *n* = 2802 (%)	≥ 10 drugs *n* = 716 (%)	*p* values		
					1–4 vs	1–4 vs	5–9 vs
					5–9 drugs	≥10 drugs	≥10 drugs
Benzodiazepine derivatives	770	280 (3.6)	330 (11.8)	160 (22.3)	< .001^†**^	< .001^†**^	< .001^†**^
Non-benzodiazepine hypnotics	380	149 (1.9)	166 (5.9)	65 (9.1)	< .001^†**^	< .001^†**^	.002^†**^
Tricyclic antidepressants	45	20 (0.3)	15 (0.5)	10 (1.4)	.035^‡^	< .001^‡**^	.022^‡^
Sulpiride	24	7 (0.1)	10 (0.4)	7 (1.0)	.005^‡*^	< .001^‡**^	.061^‡^
Antiparkinsonian drugs (anticholinergic drugs)	35	16 (0.2)	18 (0.6)	1 (0.1)	.001^‡*^	NA	.150^‡^
Combined therapy with multiple	270	42 (0.5)	123 (4.4)	105 (14.7)	< .001^†**^	< .001^†**^	< .001^†**^
antithrombotic drugs							
(antiplatelet drugs, anticoagulants)							
Digoxin (> 0.125 mg/day)	6	0 (0.0)	3 (0.1)	3 (0.4)	.019^‡^	< .001^‡**^	.103^‡^
Loop diuretics	596	84 (1.1)	299 (10.7)	213 (29.7)	< .001^†**^	< .001^†**^	< .001^†**^
Aldosterone antagonists	409	93 (1.2)	186 (6.6)	130 (18.2)	< .001^†**^	< .001^†**^	< .001^†**^
α1-Receptor blockers	105	27 (0.3)	40 (1.4)	38 (5.3)	< .001^†**^	< .001^†**^	< .001^†**^
(nonselective for receptor subtypes)							
H_1_ receptor antagonists	49	20 (0.3)	19 (0.7)	10 (1.4)	.003^‡*^	< .001^‡**^	.065^‡^
(first generation)							
H_2_ receptor antagonists	647	234 (3.0)	301 (10.7)	112 (15.6)	< .001^†**^	< .001^†**^	< .001^†**^
Antiemetic drugs	126	35 (0.5)	68 (2.4)	23 (3.2)	< .001^‡**^	< .001^‡**^	< .001^‡**^
Sulfonylureas	173	26 (0.3)	107 (3.8)	40 (5.6)	< .001^†**^	< .001^†**^	.035^†^
Biguanides	291	68 (0.9)	163 (5.8)	60 (8.4)	< .001^†**^	< .001^†**^	.012^†*^
Thiazolidine derivatives	89	24 (0.3)	45 (1.6)	20 (2.8)	< .001^‡**^	< .001^‡**^	.043^‡^
α-Glucosidase inhibitors	212	46 (0.6)	101 (3.6)	65 (9.1)	< .001^†**^	< .001^†**^	< .001^†**^
SGLT2 inhibitors	40	9 (0.1)	25 (0.9)	6 (0.8)	< .001^‡**^	.001^‡**^	NA
Oxybutynin (oral)	3	2 (0.0)	1 (0.0)	0 (0.0)	NA	NA	NA
Muscarinic receptor antagonists	139	71 (0.9)	39 (1.4)	29 (4.1)	.035^†^	< .001^†**^	< .001^†**^
NSAIDs	836	344 (4.5)	341 (12.2)	151 (21.1)	< .001^†**^	< .001^†**^	< .001^†**^

The target PIMs are “drugs to be prescribed with special caution” to the elderly according to the Medications for the Elderly Guidelines 2015, or drugs that can be extracted from the electronic medical records (EMR) even if the target population is limited. Sliding-scale insulin was excluded because it could not be extracted from the EMR. ^†^ The *χ*^2^ test or ^‡^Fisher’s exact test was used to compare the differences between each group. Correction with the Bonferroni method was performed, and *p* < .017 (*p* < .025 for antiparkinsonian drugs and SGLT2 inhibitors) was considered significant. *p < .017 (*p* < .025); **p < .003 (*p* < .005). NA, not applicable

**Fig. 2:**
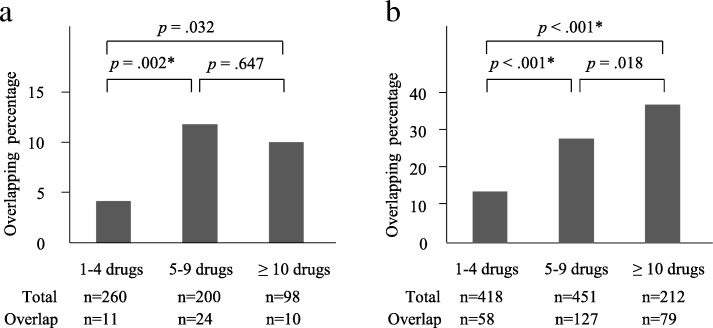
Overlapping drugs with the same mechanism of action. (a) Overlapping anticholinergic drugs (b) Overlapping benzodiazepine receptor agonists The *χ*^*2*^ test was used to compare the differences between each group (a,b). Correction with the Bonferroni method was performed, and *p* values < .017 were considered significant. *p < .017

Percentage of overlapping prescription of anticholinergics or benzodiazepine receptor agonists in the 5–9-drugs group was higher than in the 1–4-drugs group (Fig. 2). No significant difference in overlapping prescription for anticholinergics or benzodiazepine receptor agonists was found between the 5–9-drugs and 10 or more-drugs groups. Patients receiving overlapping prescriptions for anticholinergic drugs received significantly more prescriptions for laxatives [OR 2.44 (95% CI, 1.30–4.61)].

In the year following the prescription survey, 1900 (16.9%) patients in the study were hospitalized (Table 3). We identified loop diuretics and nonsteroidal anti-inflammatory drugs (NSAIDs) as independent risk factors for hospitalization [OR 1.73 (95% CI, 1.38–2.16) and 1.29 (95% CI, 1.08–1.54), respectively]. The number of PIMs was an independent risk for hospitalization [OR 1.18 (95% CI, 1.12–1.26), *p* < .001], but not the use of PIMs other than loop diuretics and NSAIDs.

**Table 3:** Results of multiple logistic- regression analysis of PIMs-related risk factors that can affect hospitalization

	Number of patients	Adjusted^†^ OR (95% CI)	*p* value
	hospitalized/Total (%)		
Age		0.99 (0.98–0.99)	.001^**^
Sex			
Male	1113/5653 (19.7)	1.56 (1.40–1.72)	< .001^***^
Female	787/5588 (14.1)		
Total number of drugs		1.06 (1.03–1.08)	< .001^***^
Number of medical departments		1.43 (1.29–1.59)	< .001^***^
Benzodiazepine derivatives	142/770 (18.4)	0.90 (0.74–1.10)	0.314
Non-benzodiazepine hypnotics	78/380 (20.5)	1.11 (0.85–1.45)	0.439
Tricyclic antidepressants	9/45 (20.0)	1.09 (0.51–2.35)	0.821
Sulpiride	2/24 (8.3)	0.36 (0.81–1.58)	0.173
Antiparkinsonian drugs	4/35 (11.4)	0.64 (0.22–1.84)	0.409
(anticholinergic drugs)			
Combined therapy with multiple antithrombotic drugs	80/270 (29.7)	1.26 (0.94–1.69)	0.116
(antiplatelet drugs, anticoagulants)			
Digoxin (> 0.125 mg/day)	1/6 (16.7)	0.27 (0.30–2.48)	0.249
Loop diuretics	198/596 (33.2)	1.73 (1.38–2.16)	< .001^***^
Alderostone antagonists	123/409 (30.1)	1.24 (0.95–1.61)	0.108
α1-Receptor blockers	20/105 (19.0)	0.74 (0.44–1.24)	0.25
nonselective for receptor subtypes			
H_1_ receptor antagonists	11/49 (22.4)	0.98 (0.49–1.98)	0.965
(first generation)			
H_2_ receptor antagonists	107/647 (16.5)	0.80 (0.64–0.99)	.044^*^
Antiemetic drugs	34/126 (27.0)	1.45 (0.96–2.19)	0.079
Sulfonylureas	27/173 (15.6)	0.71 (0.45–1.12)	0.14
Biguanides	49/291 (16.8)	0.82 (0.58–1.17)	0.274
Thiazolidine derivatives	10/89 (11.2)	0.53 (0.27–1.08)	0.079
α-Glucosidase inhibitors	45/212 (21.2)	1.04 (0.72–1.50)	0.847
SGLT2 inhibitors	4/40 (10.0)	0.48 (0.17–1.42)	0.185
Muscarinic receptor antagonists	18/139 (12.9)	0.58 (0.35–0.96)	0.036
Oxybutynin (oral)	0/3 (0)	NA	
NSAIDs	197/836 (23.5)	1.29 (1.08–1.54)	.006^**^

^†^Adjusted for age, sex, number of medical departments, and use of other PIMs. The odds ratio (OR) was calculated using logistic regression analysis. *p* < .05 was considered statistically significant. ^*^p < .05; ^**^p < .01; ^***^p < .001. NA, not applicable

## Discussion

This study showed that increases in the total number of drugs prescribed for outpatients were associated with the prescribing of more PIMs and more overlapping DSA. Previous reports on prescriptions for the elderly in Japan are limited. As this survey obtained similar results to those of other countries, increased PIMs due to multidrug use may be a common issue across countries. Presently, little information exists on the status of the prescription issuance of PIMs in the STOPP-J. Therefore, our findings may be useful for future medical care of the elderly in Japan.

Our findings showed that overlapping DSAs increased remarkably in the 5–9-drugs group compared to that in the 1–4-drugs group. We found many cases where laxatives were prescribed for patients receiving overlapping drugs with anticholinergic effects, suggesting that drug-induced constipation increased owing to the overlapping of drugs with anticholinergic effects. The proportion of overlapping DSAs was markedly higher in the 5–9-drugs group than in the 1–4-drugs group without significant difference. Kojima et al. reported that falling and other drug-related adverse events increase in elderly patients concurrently using more than 5 or 6 drugs [14, 15], and our results may explain one of these events.

We also found that the number of PIMs was an independent risk factor for hospitalization, but the use of PIMs except for loop diuretics and NSAIDs was not. The presence or absence of PIMs was reported to affect hospitalization [16, 17], and a high number of hospitalizations was indicated in patients using specific drugs, such as loop diuretics and NSAIDs [18, 19]. It was reported that the use of loop diuretics is more likely to lead to cardiac death and re-hospitalization, even after correction for differences in background factors, including the severity of heart failure [20]. Reports showed that the use of NSAIDs is the most common cause of drug adverse events in elderly people, and that the use of NSAIDs has increased [21]. We think that these reports support our results. However, all hospitalizations in our study were not the results of drug adverse events; thus, future studies are warranted.

To date, there are several reports that multidrug use is associated with adverse events and hospitalization [2, 14, 15, 22]. However, the mechanisms by which multidrug use increases these risks remain unknown. In addition, as mentioned above, PIMs affect adverse events and hospitalization. Our results clearly showed that multidrug use was correlated with increased high-risk prescriptions, i.e., the number of PIMs and overlapping DSAs. This result may be linked to increased risk of adverse events and hospitalization due to multidrug use. It was also reported that reviewing a patient’s prescription reduces the risk of re-hospitalization of the patient [23]. Therefore, reviewing high-risk prescriptions might prevent adverse events and hospitalization.

Our study had several limitations. First, our study analyzed only the drugs prescribed in our hospital. Therefore, it is possible that the results of this survey underestimated the actual state of prescription issuance. The total number of prescribed drugs was lower than that reported in other studies in Japan [12, 24]. Second, we did not examine any prescription changes during the study period. Third, we were unable to include hospitalization that occurred in other hospitals. In addition, our method could not be used to analyze, in-depth, the backgrounds of the hospitalized patients. There are several reports showing the relationship between multidrug use and frailty, decreased activities of daily living, decreased renal function, and worsening of nutritional status [24]. More knowledge can be obtained by identifying these conditions and conducting detailed analysis.

Multidrug use is often considered inappropriate under any circumstances. A more nuanced view holds that a combination of more drugs than necessary should be defined as “polypharmacy,” for distinction from appropriate multidrug combinations [25]. Our results may contribute to elucidate multidrug combinations that should be avoided.

## Conclusion

This study revealed that the number of PIMs and overlapping DSAs were higher in patients receiving multidrug treatment, and that the number of PIMs was an independent risk factor for hospitalization. Reviewing prescriptions and considering the number of PIMs and overlapping DSAs may reduce adverse events and hospitalization.

## Data Availability

All data analyzed in this study are included in this published article.

## Abbreviations

DSAs: Drugs with the same mechanism of action
EMR: Electronic medical records;
NSAIDs: Nonsteroidal anti-inflammatory drugs
OR: odds ratio
PIMs: Potentially inappropriate medications
STOPP: Screening Tool of Older Persons’ Prescriptions
STOPP-J: Screening Tool for Older Persons’ Appropriate Prescriptions for Japanese
